# Skeletal Muscle Sorbitol Levels in Diabetic Rats with and without Insulin Therapy and Endurance Exercise Training

**DOI:** 10.1155/2009/737686

**Published:** 2009-11-23

**Authors:** O. A. Sánchez, T. F. Walseth, L. M. Snow, R. C. Serfass, L. V. Thompson

**Affiliations:** ^1^Division of Pathophysiology, Department of Physiological Sciences, School of Medicine, Universidad de Oriente, Cd Bolivar, Edo Bolivar, Venezuela; ^2^Department of Radiology and Radiological Sciences, Vanderbilt University, Nashville, TN, USA; ^3^Laboratory of Physiological Hygiene and Exercise Science, School of Kinesiology, University of Minnesota, Minneapolis, MN, USA; ^4^Department of Physical Medicine and Rehabilitation, University of Minnesota Medical School, Minneapolis, MN 55455, USA

## Abstract

Sorbitol accumulation is postulated to play a role in skeletal muscle dysfunction associated with diabetes. The purpose of this study was to determine the effects of insulin and of endurance exercise on skeletal muscle sorbitol levels in streptozotocin-induced diabetic rats. Rats were assigned to one experimental group (control sedentary, control exercise, diabetic sedentary, diabetic exercise, diabetic sedentary no-insulin). Diabetic rats received daily subcutaneous insulin. The exercise-trained rats ran on a treadmill (1 hour, 5X/wk, for 12 weeks). Skeletal muscle sorbitol levels were the highest in the diabetic sedentary no-insulin group. Diabetic sedentary rats receiving insulin had similar sorbitol levels to control sedentary rats. Endurance exercise did not significantly affect sorbitol levels. These results indicate that insulin treatment lowers sorbitol in skeletal muscle; therefore sorbitol accumulation is probably not related to muscle dysfunction in insulin-treated diabetic individuals. Endurance exercise did not influence intramuscular sorbitol values as strongly as insulin.

## 1. Introduction

Diabetes mellitus (DM) is an endocrine condition characterized by hyperglycemia and is associated with vascular and neurological complications. Common components of diabetes management include insulin and physical exercise. A significant reduction in muscle strength is observed in patients with long-term type 1 diabetes mellitus (DM) with severe distal motor neuropathy playing a major role [1]. However, neuropathy does not explain the total decline in strength, suggesting that other mechanisms must be involved. One such mechanism is postulated to be increased flux of glucose through the polyol pathway, with resultant accumulation of sorbitol, and decreased ATP production [2].

In experimental diabetes, significant alterations in contractile properties of skeletal muscle have been described in [3–5], as have increases in intramuscular sorbitol [2]. Interestingly, when sorbitol formation was inhibited by giving an aldose reductase inhibitor (ARI) to streptozotocin- (STZ-) induced diabetic rats, skeletal muscle sorbitol levels were lowered, and muscle contractile properties significantly improved [2]. Therefore, sorbitol metabolism may contribute to alterations by which skeletal muscle strength is decreased in patients with long-term DM.

Insulin decreases intracellular sorbitol by deviating glucose away from the polyol pathway and metabolizing it through nonpolyol metabolic pathways. Modifications in the metabolism of glucose induced by endurance exercise training have the potential to affect the concentrations of sorbitol in diabetic individuals too. For example, endurance exercise training increases the synthesis and activity of hexokinase II [6, 7], which increases the capacity of the skeletal muscle cell to phosphorylate glucose with subsequent metabolism through glycolysis. These training-related metabolic adaptations should increase glucose utilization and reduce intracellular glucose levels, thereby decreasing glucose metabolism via the polyol pathway.

The main purpose of this study was to determine the effect of insulin treatment in STZ-induced diabetes on skeletal muscle sorbitol values. Our goal was to mimic the conditions observed in clinical settings by administering insulin to the STZ-induced diabetic rats. We hypothesized that insulin administration would significantly decrease sorbitol accumulation in skeletal muscle. A secondary purpose was to determine the benefits of endurance exercise training on skeletal muscle sorbitol accumulation in STZ-induced diabetes. We hypothesized that exercise would also significantly decrease sorbitol accumulation in skeletal muscle.

## 2. Methods

### 2.1. Animal Care and Diabetes Induction

The University of Minnesota Institutional Animal Care and Use Committee approved the experimental procedures and the Principles of Laboratory Animal Care according to NIH publications were followed. Thirty-nine male Sprague-Dawley rats (Charles River, Wilmington, MA) were used in this study. They were housed in a light (12 hour light : dark cycle) and temperature (20–22°C) controlled animal facility. Food and water were available ad libitum. In order to determine the effects of insulin, animals were randomly assigned to one of three groups: control sedentary (CS), diabetic sedentary (DS), and diabetic sedentary not insulin treated (DSNI). In order to evaluate the effects of endurance exercise animals were randomly assigned to one of four groups: CS, DS, control exercise (CE), and diabetic exercise (DE). In this portion of the study, our goal was to mimic the conditions observed in clinical settings by giving insulin to the diabetic rats. 

Diabetes was induced with intravenous administration of streptozotocin (Sigma, St. Louis, MO), 60 mg/kg of body weight, in a saline solution. Control rats were injected only with saline solution. Diabetes was confirmed if serum glucose concentration was at least 250 mg/dl on two consecutive days, with glucose testing beginning on the second day post-STZ administration. Thereafter, in the diabetic animals, serum glucose was measured three times per week for the following 12 weeks using the glucose oxidase method (Glucose Analyzer II, Beckman Instruments, Palo Alto, CA). Subcutaneous NPH insulin (Neutral Protamine Hagedorn) was administered daily to diabetic rats at the beginning of their active phase in the evening, according to a sliding scale based on serum glucose levels. Insulin was given to mimic the clinical condition of patients, and to prevent severe metabolic complications, but not to provide tight glucose control. In the diabetic rats not receiving insulin, mean glucose value was 365 ± 12 mg/dl, and in those receiving insulin mean blood glucose was 317 ± 14 mg/dl for the sedentary group and 303 ± 20 mg/dl for the exercise group (Table 1).

### 2.2. Endurance Exercise Training Program

Rats assigned to the exercise groups ran on a treadmill five times per week for 60 minutes, starting at a speed of 15 m/min, and progressively increasing speed by two to three m/min every two weeks up to a speed of 27 m/min [8, 9]. Grade was maintained at 10% for the entire training period. To avoid over training during the last six weeks of the experimental period, the speed and the duration of the daily exercise bouts were lowered from their scheduled intensity by five m/min and 10 minutes, respectively, on every other day.

To assess training status, blood lactates were determined at the end of the 12-week training period. Blood lactate was measured using a YSI lactate analyzer (YSI 2300 STAT PLUS. Yellow Springs, OH). Blood samples (20 *μ*L into a heparinzed microcapillary tube) were collected from the tail vein before and after a 10-minute treadmill running bout at a speed of 20 m/min and 10% grade. Samples were stored in Triton X and sodium fluoride until measurement. The difference between pre- and postexercise blood lactate values was determined. The blood extraction procedure took between 6 to 8 seconds to perform; therefore blood lactate values were close to the actual values during exercise. 

To assess cellular aerobic capacity in muscles with different skeletal muscle myosin heavy chain isoforms (fiber type), citrate synthase activity was determined in the white (superficial region) and red (deep lateral region) gastrocnemius muscle from the same animals using the method described by Srere P. A. [10].

### 2.3. Muscle Preparation

The exercise-trained animals were killed four to six days after their last bout of running. This timing was chosen to avoid acute effects of the last bout of exercise. At the end of 12 weeks, all groups of rats were deeply anesthetized with pentobarbital sodium (50 mg/kg of body weight i.p.). The extensor digitorun longus (EDL) and the soleus (SOL) muscles were excised, weighed, frozen in liquid nitrogen, and stored in −80°C until sorbitol determination.

### 2.4. Extraction and Determination Procedure for Sorbitol

Each muscle was cut into small pieces and ground in the presence of liquid nitrogen until it had a powdered appearance. The weight of the powered muscle was determined, after which 1 mL of ice-cold 0.5 M perchloric acid was added and the samples were sonicated to disrupt the tissue. The sonicated muscle was centrifuged for five minutes to remove precipitate. The resulting supernatant was neutralized by adding a solution containing one part of tri-n-octylamine and three parts of 1,1,2-trichlorotrifluoroethane. The solution was then vortexed for 45 seconds, then centrifuged for five minutes at 7000 rpm. The upper layer was recovered and adjusted to pH 7.0 with 2 M Tris base, and the samples were evaporated to dryness in a Speed-Vac concentrater and stored in −20°C until sorbitol determination.

The dried neutralized muscle extract was redissolved in 75 *μ*L of water and 10 uL aliquots of samples along with sorbitol standards were added to a 96 well plate. Sorbitol dehydrogenase reaction was started by adding to each well 10 uL of a reaction mix containing 20 *μ*g/*μ*L sorbitol dehydrogenase, 2 mM NAD in 50 mM glycine-NaOH buffer pH 9.7. Following incubation with sorbitol dehydrogenase for 30 minutes at room temperature, 100 *μ*L of a solution containing 65 *μ*g/mL diaphorase, 10 *μ*M flavin mononucleotide, 20 *μ*M resazurin in 50 mM glycine-NaOH, pH 9.7 was added and the fluorescence (emission at 590 nm, excitation at 544 nm) was determined on a BMG Fluorstar Galaxy fluorescent plate reader for a period of an hour. This method was sensitive enough to determine sorbitol content in the range between 10 to 750 *ρ*mol per well on the plate reader. Sorbitol content was expressed in *η*mol/mg of wet muscle weight.

### 2.5. Statistical Analysis

Descriptive statistical analysis included mean and SEM. A one way ANOVA was used to determine differences in blood lactate values and if a significant overall *F*-test was obtained Tukey HSD post hoc analysis was performed to determined differences between groups. For the citrate synthase data, there were no significant differences between diabetic and nondiabetic rats. Therefore, data were pooled and an independent *t*-test was performed between rats in the exercise and the sedentary condition for the white and red gastrocnemius muscles. 

Sorbitol data from the EDL and SOL muscles were normalized by log transformation and that data were used for statistical analysis. However, for comparison with the existing literature, sorbitol values in *η*mol/mg wet muscle weight are reported. To determine the effects of insulin on sorbitol accumulation in the EDL and SOL muscles a one way ANOVA was performed including only the DSNI, DS, and the CS groups. To determine the effects of endurance exercise training on the sorbitol content on the EDL and SOL muscles a one way ANOVA was performed between the following groups DS, DE, CS, and CE for each muscle type. When a significant *F*-test was obtained, a Tukey HSD post hoc analysis was performed. Statistical significance set at *P* < .05. All statistical analyses were performed using the statistical software program SPSS 15.0 (SPSS Inc., Chicago, IL, USA).

## 3. Results

### 3.1. General Characteristics

Table 1 summarizes the data for blood glucose, daily insulin administration, body weight, and muscle weights. Blood glucose values in the diabetic rats with and without insulin treatment were significantly higher than in CS and CE rats (*P* < .001). Blood glucose values were not significantly different between DSNI and the two other diabetic groups receiving insulin (DS, DE, *P* > .13). Significantly less insulin was used in the DE rats than in the DS rats (*P* < .05). Body weight was significantly lower in DSNI rats compared to the other experimental groups (*P* < .003). 

Muscle weights for the EDL and SOL were 72% lower in the DSNI group than in the other groups (*P* < .03). Muscle weights for the EDL and SOL were not significantly different between CS, CE, DS, and DE groups.

### 3.2. Training Induced Adaptations

**Figure 1:**
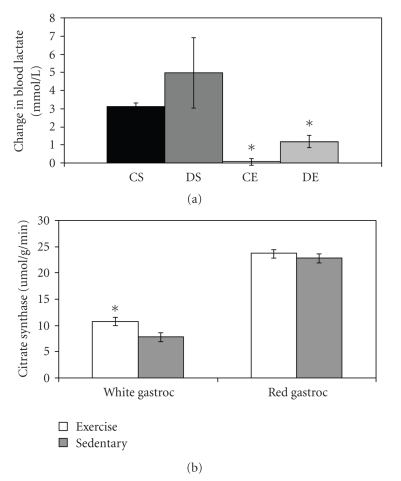
Exercise training induced adaptations. (a) Change in blood lactate values after 10 minutes of treadmill running. Values are in mmol/L ± SEM. *Exercise groups are significantly lower than sedentary groups, *P* < .03. (b) Citrate synthase activity in red and white gastrocnemius muscle. *Significantly higher than sedentary group, *P* = .015. Data from diabetic rats and nondiabetic rats were pooled because there were no significant differences between these two groups.

Following 12 weeks of endurance exercise training, the mean difference in blood lactate values between the resting state and the postacute exercise state was significantly lower in the exercised rats than in the sedentary rats (Figure 1(a), *P* < .03). A significant 28% increase in citrate synthase activity in the white gastrocnemius muscle (composed of type IIB fibers) but not in the red gastrocnemius of exercise trained rats was observed (Figure 1(b); *P* = .015). 

### 3.3. Effects of Insulin on Muscle Sorbitol Values

**Figure 2:**
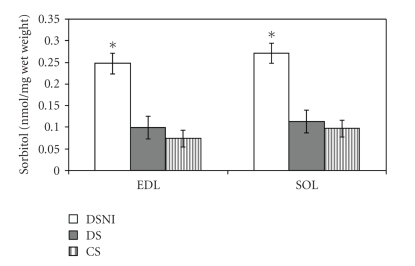
Effects of insulin administration on muscle sorbitol levels. Data are expressed as *η*mol/mg wet muscle weight ± SEM. *Significantly different from DS and CS, *P* < .01. DSNI: diabetic sedentary no insulin, DS: diabetic sedentary, CS: control sedentary, EDL: extensor digitorum longus, and SOL: Soleus.

Sorbitol levels were higher in diabetic rats not receiving insulin than in the other experimental groups (Figure 2). The magnitude of increase in both EDL and SOL was ~3 folds. Sorbitol values were not significantly different between CS and DS rats for the EDL and SOL.

### 3.4. Effects of Endurance Exercise Training on Muscle Sorbitol Values

**Figure 3:**
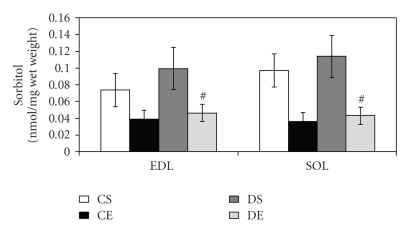
Effect of endurance training on muscle sorbitol levels. Data are expressed as *η*mol/mg wet muscle weight ± SEM. ^#^Marginally different from DS, *P* = .095. CS: control sedentary, CE: control exercise, DS: diabetic sedentary, DE: diabetic exercise, EDL: extensor digitorum longus, and SOL: Soleus.

In this study, endurance training in the diabetic rats did not reduce sorbitol levels to the degree that significance was obtained at the predetermined *P*-value of .05. However, Figure 3 shows a tendency that endurance training might be associated with a decrease in sorbitol levels in both EDL and SOL (*P* = .095). 

## 4. Discussion

### 4.1. Main Findings

This study shows the effects of insulin and endurance exercise training on sorbitol content in skeletal muscle of diabetic rats. We provide evidence that the administration of insulin to diabetic rats is sufficient to lower sorbitol content to values observed in control rats. Additionally, in insulin-treated diabetic rats, endurance exercise training showed a tendency to add to the sorbitol lowering effects of insulin although not to the preselected level of statistical significance (*P* < .05).

### 4.2. Effects of Insulin on Muscle Sorbitol Accumulation

As expected, STZ-induced diabetes increased sorbitol concentrations in both EDL and SOL muscles. This elevation was significantly lowered by insulin. Administration of insulin at a dosage that was not aimed at restoring blood glucose to normal levels, but at preventing severe metabolic complications, was sufficient to lower muscle sorbitol values in diabetic rats to values observed in nondiabetic controls. Insulin most likely exerts its sorbitol-lowering effect by increasing the activity of hexokinase II, an insulin-dependent enzyme [11]. Increased activity of hexokinase II may increase shuttling of glucose through glycolysis and glycogen storage pathways rather than through the polyol pathway. Evaluating hexokinase II activity within the same experimental paradigm is required to test the proposed hypotheses. 

With diabetes there is muscle impairment, but the underlying mechanisms remain unknown. The hypothesized potential mechanisms to explain this impairment include: diabetic motor neuropathy [12, 13], glycation of contractile proteins [14, 15], and accumulation of sorbitol [5, 14, 16–19]. Impairments in contractile parameters were observed in muscles of noninsulin treated diabetic rats [2, 4, 5], as well as in insulin-treated diabetic rats [20]. The results of the present study would suggest that sorbitol accumulation is not contributing to the observed muscle dysfunction when insulin is administered to diabetic rats.

### 4.3. Effects of Endurance Exercise Training on Muscle Sorbitol Accumulation

Endurance exercise training appears to lower sorbitol values in the two muscles investigated in this study (EDL and SOL). The mechanism responsible for lowering sorbitol is probably related to the acute and long-term effects of exercise on glucose metabolism. The increase in citrate synthase activity and the lower blood lactate values in the endurance-trained rats are evidence that metabolic adaptations occurred secondary to the endurance training protocol. Such adaptations have previously been described to include an increase in hexokinase II activity that may divert glucose from the polyol pathway [6, 21, 22]. As stated previously, further studies are required to tease out the specific roles of hexokinase II and the polyol pathway.

### 4.4. Other Possible Biochemical Mechanisms for Muscle Dysfunction Related to the Polyol Pathway

The findings in the current study suggest that sorbitol accumulation does not appear to be responsible for the functional alterations observed in muscles of diabetic rats. An alternative explanation could be that the polyol pathway induces alterations in the redox state of the muscle, increasing deleterious oxidative stress [23–25]. Thus, high intracellular sorbitol concentrations may be a biomarker for an alternative mechanism that remains to be elucidated [5, 26].

### 4.5. Limitations and Implications

Rats in this study were killed four–six days after their last bout of exercise. This timing might have influenced the results because the acute effects of exercise on glucose uptake and metabolism lasts at most for 40 hours [27, 28]. We suspect that the effect of endurance exercise-training on lowering muscle sorbitol levels might have been of greater magnitude if the muscles would have been dissected closer in time to the last bout of exercise. Accordingly, we might have missed a transient statistically significant difference in sorbitol values in the EDL and SOL muscles of endurance exercised trained rats when compared to the sedentary groups.

In summary, we have determined that insulin treatment of diabetic rats significantly reduces skeletal muscle sorbitol concentration. Moderate-to-high intensity endurance exercise training may further decrease sorbitol accumulation, but results are less conclusive. Therefore, the results of this study seem to indicate that sorbitol accumulation is most likely not related to skeletal muscle dysfunction in insulin-treated diabetes.

## Figures and Tables

**Table 1:** Mean blood glucose, dose of NPH daily insulin administration daily, and final body weight by group. Data are expressed as means ± SEM. *Significantly different from CE and CS. ^*φ*^Significantly different from DS. ^#^Significantly different from CE, CS, DE, and DS groups. ^*ψ*^Significantly different from all other groups. EDL: extensor digitorum longus, SOL: Soleus.

Groups (*n*)	Blood glucose (mg/dl)	NPH Insulin (IU)	Body weight (g)	EDL (g)	SOL (g)
Diabetes sedentary no. insulin, DSNI (6)	365 ± 12*	—	372 ± 20^#^	0.157 ± 0.03^*ψ*^	0.169 ± 0.04^*ψ*^
Diabetic sedentary, DS (9)	317 ± 14*	2.32 ± 0.04	504 ± 29	0.226 + 0.03	0.224 ± 0.03
Diabetic exercise, DE (8)	303 ± 20*	2.09 ± .05^*φ*^	451 ± 11	0.224 ± 0.02	0.234 ± 0.03
Control sedentary, CS (8)	120 ± 3	—	549 ± 21	0.251 ± 0.03	0.241 ± 0.04
Control exercise, CE (8)	119 ± 4	—	509 ± 21	0.253 ± 0.03	0.238 ± 0.03
